# Effective Magnetic Field Dependence of the Flux Pinning Energy in FeSe_0.5_Te_0.5_ Superconductor

**DOI:** 10.3390/ma14185289

**Published:** 2021-09-14

**Authors:** Masood Rauf Khan, Antonio Leo, Angela Nigro, Armando Galluzzi, Massimiliano Polichetti, Valeria Braccini, Matteo Cialone, Mario Scuderi, Gaia Grimaldi

**Affiliations:** 1 Physics Department “E. R. Caianiello”, University of Salerno, Via Giovanni Paolo II 132, I-84084 Fisciano, Italy; mkhan@unisa.it (M.R.K.); anigro@unisa.it (A.N.); agalluzzi@unisa.it (A.G.); mpolichetti@unisa.it (M.P.); 2 CNR SPIN Salerno, Via Giovanni Paolo II 132, I-84084 Fisciano, Italy; gaia.grimaldi@spin.cnr.it; 3 CNR SPIN Genova, c.so F. M. Perrone 24, I-16152 Genova, Italy; valeria.braccini@spin.cnr.it (V.B.); matteo.cialone@spin.cnr.it (M.C.); 4 CNR IMM Catania Headquarter, Strada VIII n.5 Zona Industriale, I-95121 Catania, Italy; mario.scuderi@imm.cnr.it

**Keywords:** iron-based superconductors, pinning properties, flux pinning energy

## Abstract

The role of a layered structure in superconducting pinning properties is still at a debate. The effects of the vortex shape, which can assume for example a staircase form, could influence the interplay with extrinsic pinning coming from the specific defects of the material, thus inducing an effective magnetic field dependence. To enlighten this role, we analysed the angular dependence of flux pinning energy *U*(*H*,*θ*) as a function of magnetic field in FeSe_0.5_Te_0.5_ thin film by considering the field components along the *ab*-plane of the crystal structure and the *c*-axis direction. *U*(*H*,*θ*) has been evaluated from magneto-resistivity measurements acquired at different orientations between the applied field up to 16 T and FeSe_0.5_Te_0.5_ thin films grown on a CaF_2_ substrate. We observed that the *U*(*H*,*θ*) shows an anisotropic trend as a function of both the intensity and the direction of the applied field. Such a behaviour can be correlated to the presence of extended defects elongated in the *ab*-planes, thus mimicking a layered superconductor, as we observed in the microstructure of the compound. The comparison of FeSe_0.5_Te_0.5_ with other superconducting materials provides a more general understanding on the flux pinning energy in layered superconductors.

## 1. Introduction

For high-power applications of superconductors, vortex pinning is a fundamental aspect to be taken into consideration since it regulates the critical current density *J*_c_. This is the highest electric current density that can flow in a superconductor without dissipation, i.e., raising the current density beyond *J*_c_ causes the vortices to move and the lossless regime vanishes. Vortex pinning is intimately connected to the structure of the defects into the material and the characteristics of the vortex matter in a real superconductor [1,2]. Iron-based superconductors (IBS) have received a lot of attention across the world, reminding us as to what happened when high-*T*_c_ cuprate superconductors were discovered. The high upper critical field and low anisotropy suggest that there is potential for applications. Moreover, among the different families of IBSs, Fe(Se,Te) has the lowest anisotropy with the simplest crystallographic structures, and no poisonous elements, making it more appealing than other IBSs. However, a better knowledge of the pinning process and vortex state characteristics in this material is required to enhance its *J*_c_ [3,4]. In the meantime, it has been demonstrated that the fabrication of Fe(Se,Te) coated conductors with high performances in high magnetic fields is feasible [5,6].

The pinning regime observed in the IBS might be connected to the material’s electrical anisotropy, as well as to the type of the structure’s defects [7]. Indeed, the role of the layered structure needs to be clarified, especially in comparison with high temperature superconductors (HTS), since it has a significant impact on the features of the vortex landscape [1,2]. Superconductivity is highly anisotropic in materials that have a weak coupling interaction between the layers, and the vortex line is very elastic and easily deformed, as in the case of Bi_2_Sr_2_Ca_n-1_Cu_n_O_2n+4+x_ (BiSCCO) for example [8]. On the other hand, the superconductor exhibits a less deformed vortex structure when there is strong coupling between layers, as it is for YBa_2_Cu_3_O_7-x_ (YBCO) [2]. Subsequently, the flux pinning energy dependence on the applied magnetic field has been critically studied in the past for these HTS materials [9,10]. In particular, the tilt of the magnetic field also can influence the interaction among vortices in superconductors that are not too anisotropic. When the properties of the superconductor are anisotropic, a current dispersion induced by tilted magnetic fields is produced. Supercurrents then circulate on complex paths, which consist of ellipsoids whose shape depends on the tilt of the magnetic field with respect to the crystalline direction and this easily leads to the appearance of a minimum in the interaction potential between vortices [11,12,13]. Therefore, despite the low anisotropy of Fe(Se,Te) [14], the investigation of the flux pinning energy behaviour under the influence of a magnetic field applied at different angles for IBS becomes of great interest, since the control of dissipation remains a fundamental requirement for implementing IBS in high-power applications.

## 2. Experimental Details

Several microbridges were patterned by standard UV lithography on 100 nm thick films of Fe(Se,Te). These films have been grown on a CaF_2_ substrate by pulsed laser deposition using a Nd:YAG laser at 1024 starting from a target whose nominal composition is FeSe_0.5_Te_0.5_, as previously described [15]. The actual film composition is Fe_0.98_Se_0.67_Te_0.33_ and it results in a critical temperature *T*_c_ = 18.5 K as estimated by the 50% of the normal state resistance criterion. In order to estimate the angular behaviour of the flux pinning energy, the sample has been mounted on a double axis rotating platform in a Cryogenic Ltd. CFMS Cryogen-Free Measurement System. The sample orientation has been changed with respect to the fixed direction of the applied magnetic field, while the current flow direction always remains perpendicular to the field. The variable *θ* is the rotation angle formed by the applied magnetic field and the sample’s crystallographic structure, such as *θ* = 0° is for an applied magnetic field parallel to the *ab*-plane and *θ* = 90° for an applied magnetic field parallel to the *c*-axis. Flux pinning energy values have been estimated from resistance versus temperature *R*(*T*) curves acquired by a standard 4-probe technique. These measurements were performed for angles ranging from −9.5° to 100° degrees and magnetic field values up to 16 T.

Transmission Electron Microscopy (TEM) characterization has been carried out on a probe Cs-corrected JEOL JEM-ARM200F, equipped with a cold Field-emission Electron Gun (FEG) operated at 200 keV by using Selected-Area Electron Diffraction (SAED), conventional TEM diffraction contrast and Scanning Transmission Electron Microscopy STEM Z-contrast imaging techniques. The STEM micrograph was acquired by using a probe convergence semi-angle of 33 mrad with the Annular Dark-Field (ADF) detector collecting signals at a high inner semi-angle (80 mrad). Under these conditions, the observed intensities in the STEM images are proportional to the atomic number Z^1.7^ (Z-contrast imaging) [16,17].

## 3. Angular Dependence of Flux Pinning Energy

**Figure 1:**
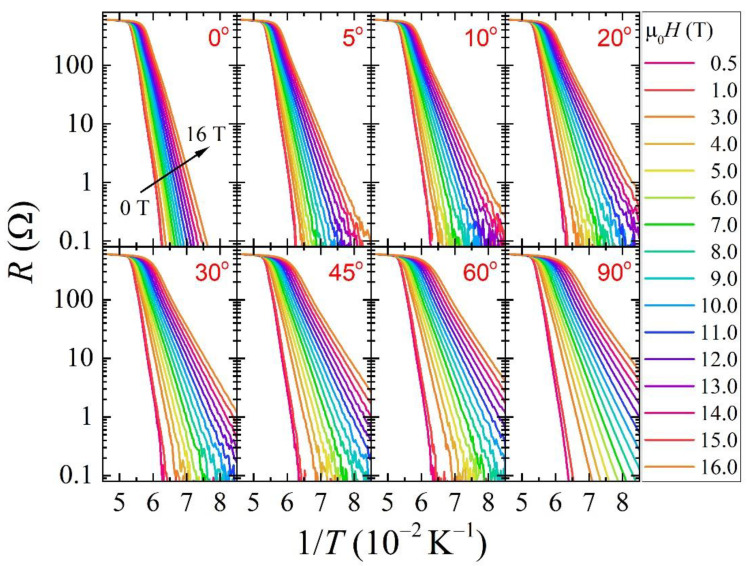
A selection of the acquired *R*(*T*) at different applied magnetic fields for the different field direction are displayed in Arrhenius plots.

**Figure 2:**
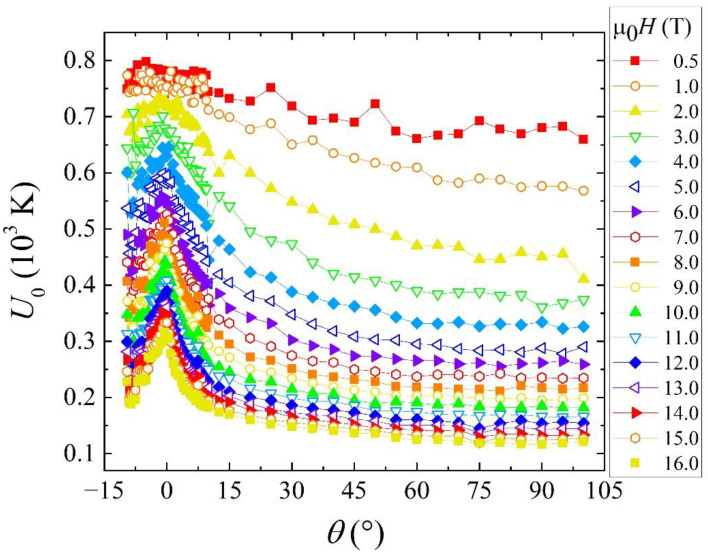
Flux pinning energy as a function of the angle *θ* up to µ_0_*H* = 16 T.

The resistance of a superconductor below the transition can be written in the form *R*(*T*,*H*,*J*) = *R*_0_exp[*U*(*T*,*H*,*J*)/k_B_*T*] where *U*(*T*,*H*,*J*) is the flux pinning energy and k_B_ is the Boltzmann constant. The resistance as a function of 1/*T* in a log plot (Arrhenius plot) related to some of the *R*(*T*) curves acquired at different applied magnetic field values and different orientation *θ* between the field and the sample are shown in Figure 1. We are reminded that the flux pinning energy is directly related to the slope of a straight line drawn on Arrhenius curves in the region of resistance lower than 10% of the normal state of resistance. All the curves have been acquired with a fixed bias current of 10 μA. The resistive transition at a fixed magnetic field value broadens as the angle with respect to the *ab*-plane increases. This angular dependence seems to have an anisotropic behaviour that is reflected in an equally anisotropic behaviour of the flux pinning energy (see Figure 2). Such an angular dependency is not observed by just evaluating *U*(*H*) at **H**//*ab* and **H**//*c*, as reported for instance in β-FeSe single crystals [18]. Therefore, angular measurements provide a more detailed picture of the behaviour of the flux pinning energy. Here we estimate the flux pinning energy as a function of the applied magnetic field through the analysis of the *R*(*T*) curves by following the Tinkham’s approach. In this model, the pinning activation can be factorized *U*(*T*,*H*,*J*) = *U*_0_(*H,J*)g(*t*), where g(t)=(1−t2)(1−t4)n2, and $t=\frac{T}{T_{c}}$ is the reduced temperature [19,20]. The *n*-exponent in the g(*t*) function is usually set to 1 in the case of High Temperature Superconductors, thus we consider g(t)=(1−t2)(1−t4)12.

Figure 2 shows the flux pinning energy as a function of *θ* at different applied magnetic fields. By increasing the applied magnetic field from 0 T to 16 T, along the *ab*-plane orientation, the flux pinning energy tends to drop from ~800 K to ~310 K, while along the *c*-axis direction, it tends to drop from ~680 K to ~120 K. These flux pinning energy values are comparable to those reported for other IBSs, such as β-FeSe single crystals [18], Fe_1.06_Te_0.6_Se_0.4_ [21] and Fe(Te,S) single crystals [22]. We note that a different choice of *n*-exponent in the g(*t*) function can lead to *U*_0_ values a factor of 3 lower than those evaluated in the case *n* = 1 [23]. On the other hand, by using a different approach, as the modified Thermally Activated Flux-Flow (TAFF) model [24], the estimated *U*_0_ values could be higher by a factor of 5. It has been proven that the choice of the approach does not significantly affect the overall trend of *U*_0_(*H*) [21,23].

## 4. Microstructure Analysis of Material Defects

**Figure 3:**
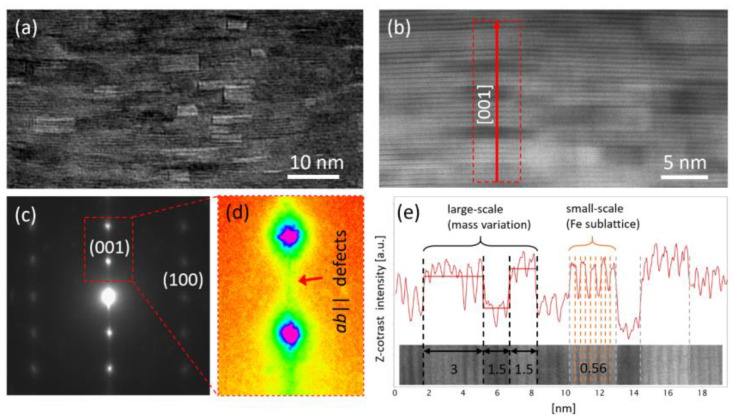
(**a**) Diffraction contrast TEM image; (**b**) STEM Z-contrast image; (**c**,**d**) SAED image from (**a**); (**e**) Z-contrast intensity profile along the red line marked in (**b**).

A TEM analysis has been performed in order to achieve a better knowledge of the material defects that can act as pinning centers. Figure 3 shows the cross-section of the FeSe_0.5_Te_0.5_ film in which many elongated rectangular shaped grains parallel to the *ab* orientation are observed in both the TEM diffraction contrast (see Figure 3a) and STEM Z-contrast (see Figure 3b). Indeed, Figure 3a underlines the presence of material defects. In particular, the elongated shape of the spots in the SAED images in Figure 3c,d, marks the prevalence of defects parallel to the *ab* planes. Moreover, the Z-contrast intensity profile along the [001] direction of Figure 3e shows oscillations evidencing a small- and large-scale *ab* layered structure. In particular, the large scale structure, made up by layers of 1 to 3 nm thickness, originates from the stoichiometry variations of the different crystallographic *ab*-domains composing the film [25], whereas the small scale structure is typically constituted by the naturally layered crystallographic structure of the Fe(Se,Te) compound.

## 5. Correlation between Nanoscale Defects and Pinning Energy

The presence of an increasing remarkable peak in the *U*_0_(*H,θ*) behaviour reported in Figure 2 deserve a more detailed analysis. The question that arises is if such a peak resembles the one observed in HTS layered materials or not.

At higher fields, the vortex spacing becomes substantially smaller than the penetration depth, resulting in a drop of the flux pinning energy value. The flux pinning energy is substantially higher for the magnetic field applied parallel to the *ab*-plane, i.e., *θ* = 0°, and then monotonically decreases, rather than for all the other magnetic field orientations. It is conceivable that the coupling strength between the Fe*Ch* planes, which affects the pinning behaviour, is more important than the defect structure itself [8,18]. In other IBSs, similar flux pinning energies for **H**//*ab* and **H**//*c* were reported [21,22,26]. In another case, for the FeSe compound, it is estimated at 16 T, by Amigo et al. [27], that the flux pinning energy for **H**//*ab* is substantially greater than for **H**//*c* due to the presence of only point defects. Moreover, the presence of additionally correlated defects is revealed by extra peaks in the *U*_0_(*H*,*θ*) at specific angular orientations of the applied field [27]. In the case of FeSe, such correlated defects serve as vortex pinning centers. By Te-doping these defects disappear, and there is no apparent sign of them in the angular dependence of the flux pinning energy as well [27]. Nevertheless, evaluating the angular pinning energy for K_0.8_Fe_1.65_Se_2_ crystals, M.L. Teng et al. found a dip along the *ab*-plane orientation due to the formation of kinks in the vortices. This has been related to the possible reduction of intrinsic pinning at a certain temperature because intrinsic pinning due to the layered structure dominates at lower temperatures [28].

We observed a different behaviour in the Fe(Se,Te) compound. In our case, the pinning mechanism could come from extended defects that are elongated along the *ab*-plane, but it may also be attributed to the layered structure. In fact, whenever the magnetic field is applied along the *ab*-plane a strong increase in flux pinning energy is observed, and this peak increases as a function of the magnetic field intensity, as shown in Figure 2. Iida et al. also observed a comparable behaviour in the Fe(Se,Te) at high magnetic field intensity from 1 T to 9 T with a peak along the *ab*-plane corresponding to an *θ* = 0° orientation [29]. A similar peak at *θ* = 0° for the *U*_0_(*H*,*θ*) highlights the ab-pinning ability of Fe(Se,Te) films due to the defect structure along the ab-plane observed in the microstructure by TEM analysis, as already found by the angular dependence of another pinning-related physical quantity, that is the pinning force [25]. However, at fields less than 1 T, we measured an isotropic behaviour of the flux pinning energy *U*_0_(*θ*), while at higher fields above 2 T, an anisotropic behaviour has been observed and reported in Figure 2. At low magnetic fields, the spacing between the vortices is larger than the average separation of the extended defects so vortices are not interacting between each other, and the behaviour results isotropic. As the intensity of the magnetic field increases, the interaction among the vortices increases and the resulting collective response strengthens the pinning interaction with the defects, which can be responsible for the observed anisotropy.

## 6. Magnetic Field Dependence of Flux Pinning Energy

**Figure 4:**
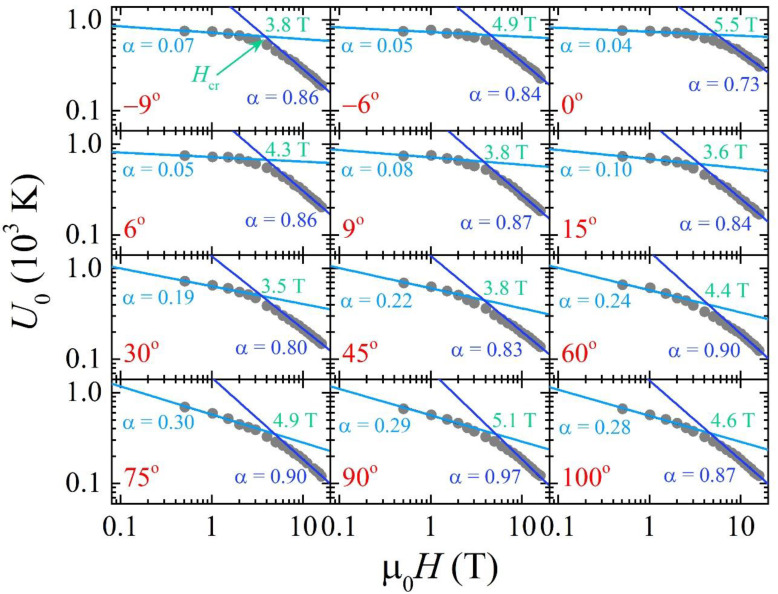
Flux pinning energy as a function of the applied magnetic field in the different field configurations.

**Figure 5:**
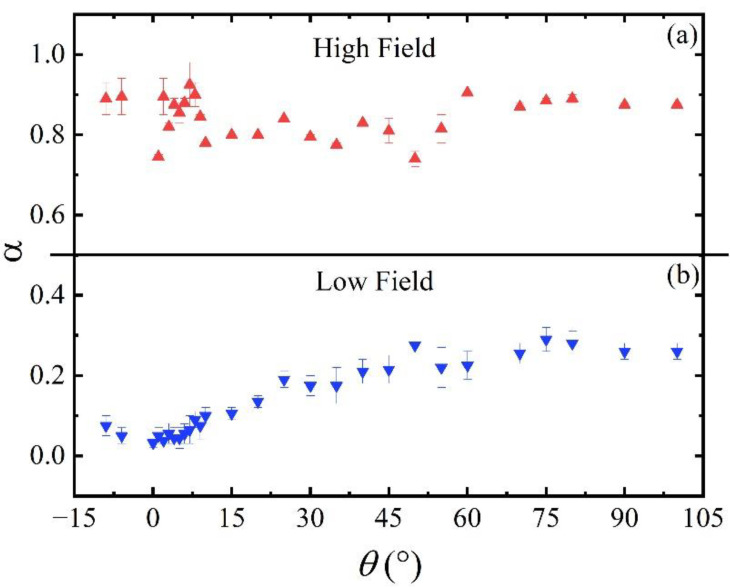
Values of α in (**a**) high and (**b**) low field ranges.

Besides material defects, we can analyse the field dependence of the flux pinning energy to clarify the pinning mechanism. In particular, it exhibits the expected power law *U*(*H*) = C*H*^−α^ behaviour, where α can assume different values depending on the dominant pinning regime. On a log-log scale, the flux pinning energy as a function of the applied magnetic field for the film under examination is shown in Figure 4. A linear fit has been performed on each *U*(*H*) curve with α and C as fitting parameters in the low field region from 0.5 T to 2 T, and in the high field region from 10 T up to 16 T. In the low field region, the exponent α shows a monotonous increase from the 0.04 minimum value at *θ* = 0° to the maximum value of 0.29 at *θ* = 90°. In the case of a high field region α shows a fluctuating behaviour around a constant value of 0.85 as reported in Figure 5.

In the low field range, the Fe(Se,Te) compound demonstrates modest power law dependency with α ≤ 0.3, while in the higher field region α is always higher than 0.5, similar to the behaviour previously reported [23,30,31]. According to the literature, a single vortex pinning regime can be associated to an almost field independent *U*_0_, i.e., α ≈ 0 [1], while for *α >* 0.5 one deals with a collective vortex pinning regime [32]. In the range where it results 0 ≈ α < 0.5, we can presume that a single-vortex like pinning regime is achieved, which becomes less and less effective as α approaches the 0.5 value due to increasing vortices interactions. In Figure 4 we marked as crossover value *H_cr_* the cross between the fitting lines in the low and high field regions. This value reduces from 5.45 T to 3.54 T when the angle between the applied magnetic field and the sample increases up to θ=30°. After that, the crossover field began to escalate from 3.83 T to 5.11 T up to θ=90°. The *H_cr_* value almost around 5 T results for **H**//*ab* and **H**//*c*, and this is in agreement with the Fe(Se,Te) and Fe(Te,S) single crystals [21,22]. Such a behaviour of the crossover field can be interpreted as a coexistence of a single vortex pinning regime and collective pinning regime in an extended field range. In fact, instead of a net change between the field independent trend with α ≈ 0 and the expected power law behaviour with a finite α value, there is a smooth rounding of the *U*_0_(*H*) expanding around the crossover region.

The density of acquired data reveals that the *U*_0_(*H*) crossover is more gradual then usually seen in previous works. Thus, it is difficult to identify proper linear regions and enlarging/narrowing the range can significantly change the α estimation results, as it happens for example in the case of [33]. Also, the model used to evaluate the *U*_0_(*H*) values can affect the value of α obtained by the fitting procedure [21]. Different pinning properties can also lead to different estimated α and crossover points. However, it is important to stress that the general picture for Fe(Se,Te) thin films is always shown, that is the presence of a gradual crossover from a single to a collective vortex pinning regime at field values of few teslas.

The angular dependence of α in the low field range clearly shows a trend, as displayed in Figure 5b, once the field orientation varies from **H**//*ab* (i.e., *θ* = 0°) to **H**//*c* (i.e., *θ* = 90°) while keeping its value fixed. Different explanations can be given for this behaviour, as for example, an effective field component along one direction or elongated pinning sites which act differently depending on the field direction.

**Figure 6:**
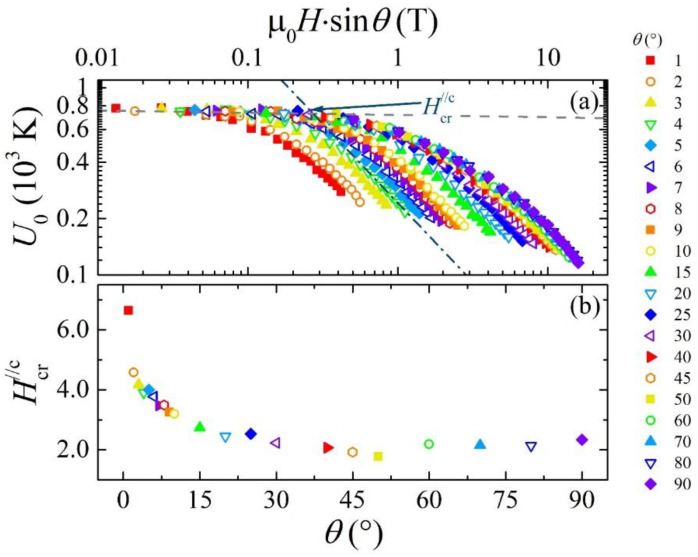
(**a**) Flux pinning energy for several magnetic fields and angles as a function of the perpendicular component of the applied field. (**b**) The crossover field perpendicular component at all measured angles.

To investigate the α trend in the low field region and, more in general, the angular dependence of the flux pinning energy, a first attempt would be following the scaling approach by Blatter et al. [34]. In this approach, the scaling factor is given by ε^2^(*θ*) = γ^−2^·cos^2^(*θ*) + sin^2^(*θ*), with γ as the anisotropy factor, which in our case can be estimated in 1.3 at 0 K [14]. Unfortunately, this scaling does not agree with the *U*_0_(*H*,*θ*) data for *θ* less than approximately 30°. Interestingly, the 30° value is equivalent to the 60° value above which Llovo et al. observe a deviation of the *H*_c2_(*θ*) from the ε^2^(*θ*) scaling [35]. A further step is to test the scaling approach followed by Xiao et al. in the case of the layered HTS BiSCCO compound [36], an approach which already has been proven effective to describe the angular dependence of another physical quantity strongly dependent on material pinning properties, which is the Flux Flow Instability critical voltage V* [37]. Thus, we plot the *U*_0_ values at different angles and fields as a function of the perpendicular component μ_0_*H*sin(*θ*) in Figure 6a.

We find that not all data fall on the same curve. The missing scaling is a signature of the fact that the anisotropy of the material is very weak [37], and this contrasts with the high temperature superconductor’s behaviour [38]. In any case, it seems that a partial scaling can be achieved above 20°, when the crossover field’s perpendicular component becomes insensitive to the angular variation of the applied field, as reported in Figure 6b. A similar behaviour was also observed in the HTS materials [38,39,40]. For example, in the YBCO based multilayers [38], wherever the scaling with perpendicular components works above the 6° value is probably due to higher values of anisotropic parameters, thus the scaling works above such a low angle. In our case, indeed, the scaling works above a much larger angle of 20°. In fact, the flux pinning energy shows a very weak field dependent behaviour as the *U*_0_ changes very slowly with respect to μ_0_*H*sin(*θ*) up to 0.5 T. This gives an indication that the single vortex pinning behaviour (i.e., α ≈ 0) is dominating when *θ* is larger than 20°. On the other hand, the activation energy *U*_0_ shows a strong field dependent behaviour (i.e., larger value of α) for μ_0_*H*sin(*θ*) > 1 T. According to previous findings, it can be argued that if the scaling is followed then the presence of uncorrelated pinning centres is generally expected [1]. In our case, the scaling does not operate throughout the whole angular range, indicating that both types of correlated and uncorrelated pinning centres can be present.

## 7. Conclusions

In conclusion, we investigated the angular applied magnetic field dependency of flux pinning energy in Fe(Se,Te) thin films grown on a CaF_2_. The flux pinning energy is a decreasing function as the applied magnetic field increases. When the applied magnetic field is parallel to the *ab*-plane orientation that is θ = 0°, the flux pinning energy is much larger as compared to all the other orientations. With increasing θ, from the *ab*-plane to the *c*-axis, i.e., *θ* approaching 90°, the flux pinning energy decreases, thus reaching the minimum value. Such a peaked behaviour of the *U*_0_(*θ*,*H*) for *θ* = 0° reminds us of the behaviour observed in the layered HTS. Based on the microstructure analysis, *ab*-oriented defects are identified by TEM, so that the observed behaviour of the flux pinning energy can be ascribed not only to the naturally layered structure of the superconducting material, but also to the presence of elongated defects parallel to the layered orientation, which may act as correlated pinning centres.

## Data Availability

The data underlying this article will be shared on reasonable request from the corresponding author.
